# Role of *Anopheles* Mosquitoes in Cache Valley Virus Lineage Displacement, New York, USA

**DOI:** 10.3201/eid2802.203810

**Published:** 2022-02

**Authors:** Constentin Dieme, Kiet A. Ngo, Shaun Tyler, Joseph G. Maffei, Steven D. Zink, Alan P. Dupuis, Cheri A. Koetzner, Chelsea Shultis, Jessica Stout, Anne F. Payne, P. Bryon Backenson, Lili Kuo, Michael A. Drebot, Alexander T. Ciota, Laura D. Kramer

**Affiliations:** New York State Department of Health, Slingerlands, New York, USA (C. Dieme, K.A. Ngo, J.G. Maffei, S.D. Zink, A.P. Dupuis, C.A. Koetzner, C. Shultis, J. Stout, A.F. Payne, L. Kuo, A.T. Ciota, L.D. Kramer);; Public Health Agency of Canada, Winnipeg, Manitoba, Canada (S. Tyler, M.A Drebot);; New York State Department of Health, Albany, New York, USA (P.B. Backenson);; State University of New York at Albany, Albany (A.T. Ciota, L.D. Kramer)

**Keywords:** Cache Valley virus, viruses, mosquitoes, Anopheles quadrimaculatus, Anopheles punctipennis, surveillance, lineage displacement, vector competence, vector-borne infections, zoonoses, New York, United States

## Abstract

Cache Valley virus (CVV) is a mosquitoborne virus that infects livestock and humans. We report results of surveillance for CVV in New York, USA, during 2000–2016; full-genome analysis of selected CVV isolates from sheep, horse, humans, and mosquitoes from New York and Canada; and phenotypic characterization of selected strains. We calculated infection rates by using the maximum-likelihood estimation method by year, region, month, and mosquito species. The highest maximum-likelihood estimations were for *Anopheles* spp. mosquitoes. Our phylogenetic analysis identified 2 lineages and found evidence of segment reassortment. Furthermore, our data suggest displacement of CVV lineage 1 by lineage 2 in New York and Canada. Finally, we showed increased vector competence of *An. quadrimaculatus* mosquitoes for lineage 2 strains of CVV compared with lineage 1 strains.

Cache Valley virus (CVV; family *Peribunyaviridae*, genus *Orthobunyavirus*) belongs to the order *Bunyavirales*, which consists of 12 families and 46 genera that are major human, livestock, and plant pathogens (*1*). CVV contains negative-sense, single-stranded RNA organized into 3 separate segments designated large (L), medium (M), and small (S) (*2*). The L RNA segment encodes the RNA-dependent RNA polymerase (L protein); the M segment encodes 2 glycoproteins, Gn and Gc, which are inserted in the viral membrane, plus a nonstructural protein; and the S segment encodes the nucleocapsid protein and a second nonstructural protein (*3*).

CVV isolates fall into 2 lineages. Lineage 1 viruses were isolated in the United States and Canada during 1956–2011, and lineage 2 consists of more recent strains from the northeastern United States (*4*). Lineage 2 was shown to have displaced lineage 1 in Connecticut, USA, during 2010–2014 (*4*). CVV is widespread throughout North and Central America and infects sheep, cattle, white-tailed deer, and humans (*4*). The virus has been isolated from >30 mosquito species in several genera; however, the principal vectors remain unknown (*5*). Accumulating evidence from surveillance suggests that *Anopheles quadrimaculatus* and *An. punctipennis* mosquitoes might be major vectors of CVV (*6*).

We performed surveillance of CVV during 2000‒2016 in New York. We also determined vector competence of *An. quadrimaculatus* for representative CVV strains.

## Materials and Methods

### Mosquito Collection

**Figure 1:**
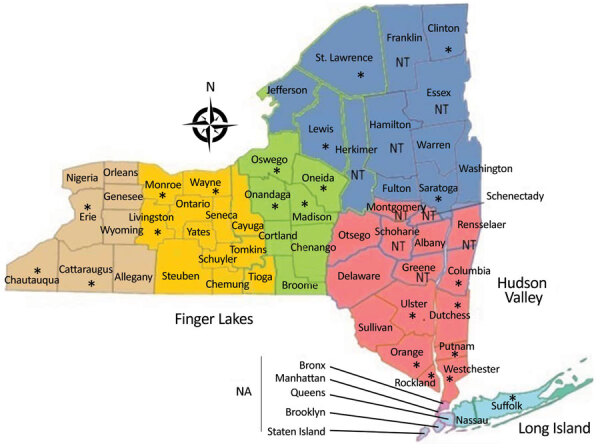
Counties in New York, USA, in which Cache Valley virus was studied during 2000‒2016 (https://www.health.ny.gov/statistics/cancer/registry/images/nycounty). Asterisks (*) indicate counties in which samples positive for Cache Valley virus were collected. NA, counties not included in data; NT, counties not tested for Cache Valley virus.

Mosquitoes were submitted from the following regions in New York: West, Finger Lakes, North, Central, Hudson Valley, and Long Island (Figure 1). Mosquitoes were collected by local health districts by using Centers for Disease Control and Prevention light traps with CO_2_ (*7*) or gravid (*8*) traps. We identified mosquitoes to species morphologically (*9*), and pooled females into groups of ≈50 mosquitoes by trap type, date collected, and trap location. Mosquitoes were transported on dry ice to the Arbovirus Laboratories, Wadsworth Center, New York State Department of Health (Slingerlands, NY, USA), for testing and were stored at −80°C until processed.

### Virus Isolation

We processed mosquito pools as described (*10*,*11*). In brief, we homogenized pools in 1 mL of mosquito diluent containing 20% fetal bovine serum, 50 μg of streptomycin/mL, 50 U of penicillin, and 2.5 μg of amphotericin B/mL in phosphate-buffered saline in a Retsch Mixer Mill (https://www.retsch.com) set to 24 cycles/s for 2 min. We used viral stocks of 2 CVV strains isolated from cerebrospinal fluid of humans (strain Hu-2022) and from brain tissue (strain PA) (*12*,*13*) for RNA extraction. We used RNA extracted from brain tissues of a horse that died from neurologic disease and tested positive for CVV in this analysis. We homogenized placenta tissues from sheep (from a ewe that had given birth to a deformed lamb in a southern Ontario flock during 2011) and used them to infect Vero E6 cells for virus isolation. Cytopathic effect was observed after 6 days, and supernatant was harvested and used for RNA extraction and to generate virus stocks.

### RNA Extraction

We used extraction plates (Thermofisher, https://www.thermofisher.com), which were prepared on a Tecan Evo 150 Liquid Handler (Tecan, https://www.tecan.com) and used 50 μL of homogenates or viral stocks to extract RNA on a Magmax 96 Express (Applied Biosystems, https://www.thermofisher.com) and a MagMax Viral Isolation Kit (Thermofisher). A total of 90 μL of homogenized sample RNA was eluted.

### Primer Design and Reverse Transcription PCR

We used a standard PCR to identify CVV isolates as described (*14*). Beginning in 2012, we developed a real-time reverse transcription PCR (RT-PCR) by using new primers and probes (CVVF1, CVVR1, and CVV1 probe) to expedite the surveillance process (Table 1). A quantitative RT-PCR was developed according to manufacturer’s protocol (Quanta Biosciences, https://www.quantabio.com) with slight modifications. The final volume of the reaction was 15 μL and consisted of 10 μL of master mixture and 5 μL of template. Each reaction contained 0.7 μmol/L of each forward and reverse primers and 0.3 μmol/L of probe. We performed real-time quantitation by using ABI Prism 7500 (Life Technologies, https://www.thermofisher.com). Cycling conditions were as follows: 3 min at 50°C, followed by 10 min at 95°C, then 40 cycles of alternating 95°C for 10 s and 60°C for 30 s. After introduction of CVV lineage 2, we developed new primers and probes (CVVF2, CVVR2, and CVV2 probe) for better detection (Table 1).

**Table 1:** Sequences of primers and probes used for detection of CVV, New York, USA*

Name	Sequence, 5′ → 3′	Primer/probe
CVVF1	ACAGCCAATGGTGTCGAAAAC	Primer
CVVR1	TGCAGGGATGCTAGACAAGATG	Primer
CVV1Probe	6FAM-CTGACGGTATTGAATCAGCAT-MGBNFQ	Probe
CVVF2	GGTGCCACATAAAGAAAACTG	Primer
CVVR2	GCCAAGCAACCAAACTC	Primer
CVV-1-R	TGATGGCCAAACAACCAA AT	Primer
CVV-1-F	GTGCCACATAAAGAGAACTGGATG	Primer
CVV2Probe	56FAM-CACCCCCATCTGCTTGTTCTTTCCTGAGAG-3IBkFQ	Probe

*CVV, Cache Valley virus.

### Maximum-Likelihood Estimation

We used maximum-likelihood estimation calculations to determine prevalence of CVV in mosquitoes. These calculations were based on a program developed by Brad Biggerstaff (https://www.cdc.gov/westnile/resourcepages/mosqsurvsoft.html).

### Sequencing

We chose representative CVV samples by county, year, and species and sent them to the National Microbiology Laboratory (Winnipeg, Manitoba, Canada) for full-genome sequencing. One PCR fragment was developed for the S segment, 3 for the M segment, and 5 for L segment (Table 2), and Sanger sequencing was performed by using BigDye version 3.1 on an ABI 3730X Analyzer L (both Thermofisher). Trace files were compiled by using SeqMan II (DNAStar, https://www.dnastar.com) to get consensus sequence for each segment. Alignments were generated by using ClustalW (https://www.clustal.org) and MEGA4 software (*15*). Phylogenetic trees were generated by using the maximum-likelihood method in Geneious version 11.1.5 (https://www.geneious.com) and PhyML (http://www.atgc-montpellier.fr) with the Jukes‒Cantor substitution model. Robustness of the nodes was evaluated by performing 500 bootstrap replicates. Trees were rooted with the Fort Sherman virus S, M, and L segments (GenBank accession nos. KX100130, KX100131, and KX100132). Mean nucleotide distances between and within CVV lineages were calculated by using MEGAX software (https://berkstech.psu.edu).

**Table 2:** Primers used for Sanger sequencing of CVV, New York, USA*

Primer	Sequence, 5′ → 3′	Target
CVVM	AGTAGTGTGCTACCGATA	M segment
CVVMR5	ACTCCTGCCTGCCAGAGTGC	1–2239 bp
CVVMF4	AATGCATTCCCAGGAACAAC	M segment
CVVMR2	CCTCTAGAGTCTCATGATTA	1984–3725 bp
CVVMF6	ATCCCTGCATTAGGTGGAAT	M segment
CVVM	AGTAGTGTGCTACCGATA	2981–4464 bp
CVVrtL	CTGACCATACCCGAGAGGCTAGTAGTGTACTCCT	L segment
NLR10	CTGTTGCTCTTTTTGTCTTGATGTCTGAAG	1–1717 bp
LF3	GGGGGTATTCTCAGACCAGA	L segment
NLR7	GGATCTAAAACTATAAGCCAAAAATACTT	1482–3221 bp
NLF6	CTAAAGAAAGATGTAAGTTAAATACAGATG	L segment
LR4	CATCAGTGGGTCATTTAATA	2984–4722 bp
NLF8	ATATCAATGCGCCATTATACCTTATATC	L segment
LR2	CTGACATAAATTCGAACTTC	3986–5722 bp
LF11b	ACAAATTCGATGCTCTAAAAACAA	L segment
CVVrtL	CTGACCATACCCGAGAGGCTAGTAGTGTACTCCT	5474–6871 bp
CVVrtALL	CTGACCATACCCGAGAGGCTAGTGTGTACT	S segment
CVVs	AGTAGTGTGCTCCAC	1–922 bp

*CVV, Cache Valley virus; L, large; M, medium; S, small.

### Mosquito Vector Competence

A colony of unknown generations of *An. quadrimaculatus* mosquitoes (Orlando strain) was obtained from BEI Resources (https://www.beiresources.org) (MRA-139) and were maintained at 27°C under standard rearing conditions (27 ± 1°C, 70% relative humidity, 12:12-h light:dark photoperiod) (*16*). Freshly propagated virus supernatant from infected Vero (African green monkey kidney) cultures were harvested at 48 h after infection (multiplicity of infection ≈1.0) and diluted 1:1 with defibrinated sheep blood and 2.5% sucrose mixture without freezing. In addition to undiluted supernatant, 10-fold dilutions from 1:10 to 1:10,000 were made in C6/36 maintenance medium (Eagle minimum essential medium containing 2% fetal bovine serum heat-inactivated with 0.5 g/L of sodium bicarbonate plus 0.1 mmol/L nonessential amino acids plus 100 U/mL penicillin/streptomycin) before being mixed 1:1 with defibrinated sheep blood and a final concentration of 2.5% sucrose. Female mosquitoes (3–5 days old) were deprived of sugar for 1–2 hours and allowed to feed on CVV-defibrinated sheep blood‒sucrose mixture for 30 min in a Hemotek membrane feeding system (Discovery Workshops, https://accrington.cylex-uk.co.uk) with a porcine sausage casing membrane at 37°C. 

After feeding, females were anesthetized with CO_2_ and fully engorged mosquitoes were transferred to 0.6-liter cardboard containers and maintained with 10% sucrose at 27°C, 70% relative humidity¸ and a 12:12-h light: dark photoperiod. Infection, dissemination, and transmission assays were performed on days 6 and 15 after the infectious blood meal as described (*17*). On day 2 after feeding, because of the early time point, only infection and dissemination assays were performed. Dissemination rate is the proportion of mosquitoes with infected legs among infected mosquitoes; transmission rate is the proportion of mosquitoes with positive saliva among mosquitoes with disseminated infection. We compared infection, dissemination, and transmission rates among strains by using χ^2^ analysis, followed by Bonferroni corrections for multiple comparisons in GraphPad Prism version 7.05 (GraphPad Software, https://www.graphpad.com). We used a TaqMan real-time reverse transcription to detect CVV by using primers and probe targeting both lineage 1 and 2 (Table 1).

## Results

### CVV Surveillance

**Figure 2:**
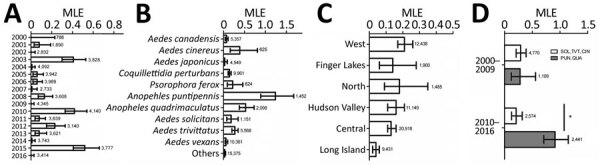
Cache Valley virus infection rate, New York, USA, during 2000‒2016, calculated by using MLE, by year (A), mosquito species (B), New York regions (C), and combined mosquito species and years (D). Error bars indicate upper and lower limits of infection rate based on 95% confidence levels. Numbers next to bars indicate number of pools tested. MLEs were calculated by using a Centers for Disease Control and Prevention resource (https://www.cdc.gov/westnile/resourcepages/mosqsurvsoft.html). *p<0.05 by χ^2^ test. CIN, *Ae.* *cinereus*; MLE, maximum-likelihood estimation; PUN, *An.* *punctipennis*; QUA, *An.* *quadrimaculatus*; SOL, *Ae.* *sollictans*; TVT, *Ae.* *trivittatus*.

We sampled 1,842,352 female mosquitoes in 57,321 mosquito pools from 2000–2016, yielding a total of 255 CVV-positive pools. We compared MLE of prevalence by year (Figure 2, panel A), mosquito species (Figure 2, panel B), and regions (Figure 2, panel C). CVV activity fluctuated substantially during the 17-year sampling period. The highest estimates of prevalence were during 2003 (0.41, 95% CI 0.30‒0.53), 2010 (0.42, 95% CI 0.33‒0.54), and 2015 (0.52, 95% CI 0.41‒0.66). No CVV was detected during 2000, 2002, 2009, and 2016. Comparable CVV prevalence was measured in 2001 (0.08, 95% CI 0.03‒0.19), 2005 (0.06, 95% CI 0.02‒0.12), 2006 (0.06, 95% CI 0.02‒0.11), 2008 (0.13, 95% CI 0.07‒0.21), 2011 (0.08, 95% CI 0.04‒0.14), and 2013 (0.08, 95% CI 0.04‒0.08).

In addition, we calculated prevalence for 10 mosquito species that had the highest number of CVV isolations. The 5 mosquito species with the highest MLE were *An. punctipennis* (1.24), *An. quadrimaculatus* (0.53), *Aedes* *cinereus* (0.39), *Ae. trivittatus* (0.27), and *Ae. sollicitans* (0.18) (Figure 2, panel B). To show which mosquito genus was driving transmission of CVV in New York, we compared the combined top 5 mosquito MLEs of *Aedes* and *Anopheles* species. The prevalence for *Ae*. *sollicitans*, *Ae. cinereus*, and *Ae. trivittatus* mosquitoes was 0.29 compared with 0.28 for *An. quadrimaculatus* and *An. punctipennis* mosquitoes during 2000–2009 (Figure 2, panel D). During 2010–2016, the combined prevalence for *An. quadrimaculatus* and *An. punctipennis* mosquitoes increased significantly to 0.91 (p<0.05 by χ^2^ test), and prevalence for *Ae. sollictans*, *Ae. cinereus*, and *Ae. trivitattus* mosquitoes decreased to 0.21. Similar infection rates were observed throughout New York regions, except Long Island, where the rate was on average lower than those for the rest of the regions (0.04) (Figure 2, panel C).

### Phylogenetic Analysis of CVV

**Figure 3:**
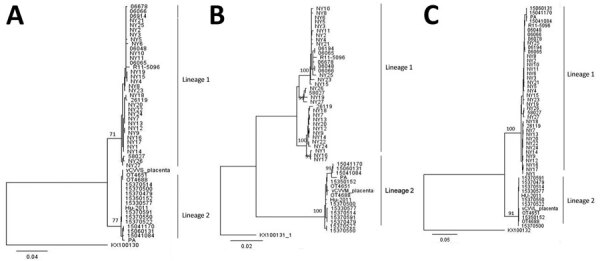
Phylogenetic analysis of Cache Valley virus, New York, USA, 2000‒2016. Maximum-likelihood phylogenetic trees show complete nucleotide sequences of small (A), medium (B), and large (C) genome segments. Numbers at nodes indicate boostrap support estimated by using 500 neighbor-joining replicates. Trees were rooted to Fort Sherman virus small, medium, and large genome segments (GenBank accessions nos. KX100130, KX100131, and KX100132). Scale bars indicate nucleotide substitutions per site.

We sequenced 48 CVV isolates representing various New York counties, hosts, and isolation dates and 3 isolates from Canada (Table 3). Most of the CVV isolates were from mosquitoes, except 4 that were isolated from 2 humans, 1 sheep, and 1 horse. Phylogenetic analysis of CVV confirmed 2 distinct lineages (lineages 1 and 2) (Figure 3). Lineage 1 contained all CVV strains obtained during 2001‒2010, and lineage 2 contained isolates obtained during 2011‒2016. Segment reassortment between M and S was observed in 4 samples, 3 from mosquito isolates (15041170, 15060131, and 15041084) and 1 from a human isolate (PA). All reassortants contain an lineage 1 L segment and lineage 2 S and M RNA segments. The 3 CVV strains isolated in Canada (1 isolate from a ewe placenta and 2 isolates from mosquito pools, all collected in Ontario during 2012) all grouped within lineage 2. There was no evidence of spatial clustering of clades within the S, M, and L segments, except the reassortants, which all came from western New York regions (Cattaraugus, Chautauqua, and Allegany Counties). Mean genetic distance calculated as the number of nucleotide substitutions per site between lineage was 0.040 for the S segment, 0.074 for the M segment, and 0.051 for the L segment (Table 4). On average, there were more nucleotide substitutions for the M segment (0.074) than for the S (0.040) and L (0.051) segments.

**Table 3:** Characteristics for Cache Valley virus strains, New York, USA, and Ontario, Canada, 2000‒2016

Year	Mosquito species	County	Strain	Lineage
2001	*Coquillettidia perturbans*	Saratoga	NY1	Lineage 1
2001	*Cq. perturbans*	Dutchess	NY15	Lineage 1
2001	*Aedes japonicus*	Ulster	NY16	Lineage 1
2001	*Cq. perturbans*	Saratoga	NY17	Lineage 1
2003	*Cq. perturbans*	Onondaga	NY2	Lineage 1
2003	*Cq. perturbans*	Oswego	NY3	Lineage 1
2003	*Ae sollicitans*	Suffolk	NY4	Lineage 1
2003	*Ae. trivittatus*	Orange	NY5	Lineage 1
2003	*Ae. trivittatus*	Westchester	NY6	Lineage 1
2003	*Ae. cinereus*	Westchester	NY7	Lineage 1
2003	*Ae. vexans*	Erie	NY8	Lineage 1
2003	*Ae. trivittatus*	Columbia	NY9	Lineage 1
2003	*Ae. trivittatus*	Dutchess	NY10	Lineage 1
2003	*Ae. vexans*	Orange	NY11	Lineage 1
2003	*Ae. canadensis*	Westchester	NY12	Lineage 1
2003	*Ae. cinereus*	Westchester	NY13	Lineage 1
2003	*Ae. triseriatus*	Orange	NY14	Lineage 1
2003	*Ae. cinereus*	Erie	NY18	Lineage 1
2003	*Ae. canadensis*	Madison	NY19	Lineage 1
2003	*Culex salinarius*	Orange	NY20	Lineage 1
2003	*Anopheles punctipennis*	Dutchess	NY21	Lineage 1
2003	*Ae. sollicitans*	Suffolk	NY22	Lineage 1
2003	*Ae. triseriatus*	Putnam	NY23	Lineage 1
2004	*Ae. vexans*	Orange	NY24	Lineage 1
2005	*Ae. vexans*	Erie	NY25	Lineage 1
2005	*Ae. vexans*	Monroe	NY26	Lineage 1
2005	*Cq. perturbans*	Lewis	NY27	Lineage 1
2006	*Ae. trivittatus*	Chautauqua	6048	Lineage 1
2006	*Ae. trivittatus*	Chautauqua	6065	Lineage 1
2006	*Ae. trivittatus*	Chautauqua	6066	Lineage 1
2006	*Ae. trivittatus*	Chautauqua	6078	Lineage 1
2006	*Ae vexans*	Chautauqua	6194	Lineage 1
2006	*An. punctipennis*	Wayne	58027	Lineage 1
2007	*An. punctipennis*	Madison	26119	Lineage 1
2011	Horse	Cattaraugus	R11–5096	Lineage 1
2011	Human	Unknown	Hu-2011	Lineage 2
2012	Sheep	Ontario	cvv_placenta	Lineage 2
2012	*Ae. trivittatus*	Ontario	OT4651	Lineage 2
2012	*An. punctipennis*	Ontario	OT4688	Lineage 2
2015	*Ae. trivittatus*	Orange	15350152	Lineage 2
2015	*Ae. vexans*	Oswego	15370591	Lineage 2
2015	*Cq. perturbans*	Onondaga	15330577	Lineage 2
2015	*Cq. perturbans*	Oswego	15370479	Lineage 2
2015	*Cq. perturbans*	Oswego	15370500	Lineage 2
2015	*Cq. perturbans*	Oswego	15370522	Lineage 2
2015	*Cq. perturbans*	Oswego	15370514	Lineage 2
2015	*Cq. perturbans*	Oswego	15370550	Lineage 2
2015	*An. punctipennis*	Cattaraugus	15041170	Reassortant
2015	*An. punctipennis*	Chatauqua	15060131	Reassortant
2015	*An. quadrimaculatus*	Cattaraugus	15041084	Reassortant
2016	Human	Allegany	PA	Reassortant

**Table 4:** Mean genetic distances for 3 genomic segments of 2 lineages of Cache Valley virus, New York, USA, 2000‒2016

Lineage	Intralineage (interlineage)		
	Small	Medium	Large
1	0.0046 (0.040)	0.0109 (0.074)	0.0062 (0.051)
2	0.0023 (0.040)	0.0033 (0.074)	0.0278 (0.051)

### Mosquito Vector Competence

We conducted vector competence assays with *An. quadrimaculatus* mosquitoes for 2 lineage 1 (NY10, NY25), 3 lineage 2 (15350152, 15330577, and Hu2011), and 4 reassortant (15041084, PA, 15041170, and 15060131) strains to determine whether there were differences between the lineages or between strains in the same lineage and to address effects of reassortment. We also hoped to determine whether vector competence was a potential mechanism of displacement of lineage 1 (Tables 5, 6). Our results indicate that lineage 1 strains are generally less infectious in *An. quadrimaculatus* mosquitoes because they had a 50% infectious dose ≈0.5–1.0 log_10_ higher than that for lineage 2 (Table 5).

**Table 5:** Relationship between dose and competence of *Anopheles quadrimaculatus* mosquitoes for Cache Valley virus, New York, USA, 2000‒2016*

Strain	Day postinfection	Blood meal titer log_10_ PFU/mL	No. infected/no. tested (%)	No. disseminated/no. tested (%)	No. transmitted.no. tested (%)
L1-NY10	15	5.1	11 /25 (44)	10/11 (90.91)	0/10 (0)
		4.2	3/25 (12)†	2/3 (66.67)	0/2 (0)
		3	0/25 (0)	NT	NT
L1-NY25	15	6.8	24/25 (96)	22/24 (91.67)	1/22 (4.55)
		5.7	13/25 (52)	12/13 (92.31)	1/12 (8.33)
		4.5	3/25 (12)†	0/3 (0)‡	NT
L2–15350152	15	5.1	18/25 (72)	18/18 (100)	0/18 (0)
		4.3	16/25 (64)	16/16 (100)	2/16 (12.50)
		3	7/25 (28)	7/7 (100)	1/7 (14.29)
L2–15330577	15	5.7	19/25 (76)	19/19 (100)	8/19 (42.11)
		5	15/25 (60)	15/15 (100)	7/15 (46.67)
		3.7	7/25 (28)	7/7 (100)	2/7 (28.57)
R-15041084	15	5.3	15/25 (60)	15/15 (100)	1/15 (6.67)
		4.4	13/25 (52)	13/13 (100)	1/13 (7.69)
		3.2	5/25 (20)	3/5 (60)	0/3 (0)
R-PA	15	4.9	10/25 (40)	0/10 (0)	NT
		3.7	6/25 (24)	0/6 (0)	NT
		2.9	0/25 (0)	NT	NT

*L1, lineage 1; L2, lineage 2; NT, not tested; R, reassortant (containing lineage 1 large RNA segment and lineage 2 small and medium RNA segments); NT, not tested. †p<0.05: lineage 1 strain infection rate compared with lineage 2 (15350152) strain when mosquitoes were fed on infectious blood ≈4–4.5 log_10_ PFU/mL titer. ‡p<0.05: lineage 1 strains dissemination rate compared with lineage 2 strains when mosquitoes were fed on infectious blood ≈4–4.5 log_10_ PFU/mL titer.

**Table 6:** Infection, dissemination, and transmission rates for *Anopheles quadrimaculatus* mosquitoes for different Cache Valley virus isolates, New York, USA, 2000‒2016*

Strain	Blood meal titer, log_10_ PFU/mL	Day postinfection	No. infected/no. tested (%)	No. disseminated/no. tested (%)	No. transmitted/no. tested (%)
L1-NY10	7.1	2	25/25 (100)	18/25 (72)†	NT
		6	42/42 (100)	42/42 (100)	5/42 (11.90)
		15	41/41 (100)	41/41 (100)	5/41 (12.20)
L1-NY25	6.6	2	18/25 (72)‡	3/18 (16.67)†	NT
		6	31/35 (88.57)‡	16/31 (51.61)†	0/16 (0)
		15	32/35 (91.43)‡	23/32 (71.88)†	4/23 (17.39)
L2–15350152	7.2	2	25/25 (100)	25/25 (100)	NT
		6	37/37 (100)	37/37 (100)	11/37 (29.73)
		15	44/44 (100)	44/44 (100)	24/44 (54.54)
L2–15330577	7.1	2	25/25 (100)	25/25 (100)	NT
		6	35/35 (100)	35/35 (100)	7/35 (20)
		15	35/35 (100)	35/35 (100)	10/35 (28.57)
L2-Hu2011	6.3	2	25/25 (100)	25/25 (100)	NT
		6	30/30 (100)	30/30 (100)	8/30 (26.67)
		15	30/30 (100)	30/30 (100)	9/30 (30)
	5.5	2	25/25 (100)	25/25 (100)	NT
		6	30/30 (100)	30/30 (100)	1/30 (3.33)
		15	24/24 (100)	24/24 (100)	5/24 (20.83)
R-15041084	7.1	2	25/25 (100)	25/25 (100)	NT
		6	41/41 (100)	41/41 (100)	6/41 (14.63)
		15	34/34 (100)	34/34 (100)	18/34 (52.94)
R-PA	5	2	10/25 (40)	4/10 (40)	NT
		6	21/35 (60)	3/21 (14.29)	0/3 (0)
		15	30/35 (85.71)	0/30 (0)	NT
R-15041170	7	2	25/25 (100)	25/25 (100)	NT
		6	30/30 (100)	30/30 (100)	1/30 (3.33)
		15	30/30 (100)	30/30 (100)	11/30 (36.67)
	4.7	2	24/25 (96)	11/24 (45.83)	NT
		6	30/30 (100)	29/30 (96.67)	0/29 (0)
		15	25/27 (92.59)	23/25 (92)	1/23 (4.34)
R-15060131	7.4	2	25/25 (100)	25/25 (100)	NT
		6	30/30 (100)	30/30 (100)	2/30 (6.67)
		15	21/21 (100)	21/21 (100)	4/21 (19.05)
	4.1	2	25/25 (100)	17/25 (68)	NT
		6	30/30 (100)	28/30 (93.33)	1/28 (3.57)
		15	29/30 (96.67)	26/29 (89.66)	2/26 (7.69)

*L1, lineage 1; L2, lineage 2; NT, not tested; R, reassortant (containing lineage 1 large RNA segment and lineage 2 small and medium RNA segments); NT, not tested. †p<0.05: lineage 1 strains dissemination rate compared with lineage 2 strains when mosquito fed on infectious blood ≈6.3–7.4 log_10_ PFU/mL titer. ‡p<0.05: lineage 1 strain infection rate compared with lineage 2 strains when mosquito fed on infectious blood ≈6.3–7.4 log_10_ PFU/mL titer.

We also found decreased dissemination and transmission for lineage 1 strains of CVV compared with lineage 2 strains (p<0.05 by χ^2^ test) (Tables 5, 6). We observed that CVV disseminated efficiently in *An. quadrimaculatus* mosquitoes by 2 days postfeeding. All mosquitoes infected with lineage 2 strains had disseminated virus, and dissemination of lineage 1 strains was more variable (Tables 5, 6). In addition, *An. quadrimaculatus* mosquitoes are a competent vector for the lineage 2 human strain but not for the human reassortant (PA) strain (lineage 1 L RNA segment and lineage 2 S and M RNA segments), which had a low dissemination rate. Except for the PA strain, *An. quadrimaculatus* mosquitoes were able to transmit CVV at day 6 postfeeding on an artificial blood meal with a high viral titer (6.0–7.0 log_10_ PFU/mL). When mosquitoes were infected with a lower viral titer (4.0 log_10_ PFU/mL), the infection rate decreased from 95%–100% to 12% for lineage 1, from 100% to 28%–64% for lineage 2, and from 85%–100% to 24%–52% for reassortants (Tables 5, 6).

## Discussion

Consistent with the findings of Armstrong et al., who analyzed CVV strains from Connecticut (*4*), we identified substantial variability in CVV activity in New York during 2000–2016. In addition, in both states, CVV could be isolated from different mosquito genera, including *Aedes, Anopheles,* and *Coquillettidia* (*6*). In our study, the prevalence of CVV in *An. punctipennis* and *An. quadrimaculatus* mosquitoes during 2010–2016 (0.91) was higher than that during 2000–2009 (0.21). Although many mosquito species are apparently infected with CVV, our data and previous surveillance data for Connecticut (*6*) all point to *Anopheles* spp. mosquitoes driving virus activity.

At least 51 different viruses have been detected in *Anopheles* spp, including 14 viruses with potential to cause febrile disease if transmitted to humans or other vertebrates, such as o’nyong nyong virus, Venezuelan equine encephalitis virus, Western equine encephalitis virus, Sindbis virus, Semliki Forest virus, Rift Valley fever virus, West Nile virus, Japanese encephalitis virus, Wesselsbron virus, Tataguine virus, Batai virus, CVV, Tahyna virus, and Tensaw virus (*18*). However, only o’nyong nyong virus, which is closely related to chikungunya virus, is known to be consistently transmitted to vertebrates by *Anopheles* mosquitoes (*19*). Other studies supported potential roles of *Anopheles* mosquito species in the transmission of Rift Valley fever virus, Mayaro virus, Eastern equine encephalitis virus, and CVV (*20**–**24*). These data and our results confirmed that *Anopheles* mosquitoes have the potential to sustain transmission cycles of arboviruses. Additional studies are needed to elucidate their role in these cycles. 

*An. quadrimaculatus* and *An. punctipennis* mosquitoes are mainly mammalian feeders in the northeastern United States, and white-tailed deer is the most commonly identified vertebrate host (*25*). Both mosquito species bite outdoors throughout the night and show higher activity at dusk and dawn and resting outdoors (*26*,*27*). In New York, white-tailed deer tested for CVV antibodies showed infection rates of 25.7% (*28*). White-tailed deer have been identified as the principal reservoir and amplification hosts for CVV, and their overabundance and availability for both *Anopheles* mosquitoes species that are frequently infected by the virus in nature (*6*,*27**–**30*) could partially explain the increase of CVV activity in *Anopheles* spp. observed in our study.

Early phylogenetic analysis of CVV strains from United States and Canada showed only a single lineage (*31**,**32*). Armstrong et al. reported emergence of a new lineage of CVV in Connecticut during 2010, displacement of lineage 1 by 2014, and no evidence of genome reassortment (*4*). Our phylogenetic analysis confirmed that the displacement of CVV lineage 1 was widespread in the region and throughout eastern Canada because the CVV lineage 2 was responsible for several outbreaks of fetal malformation disease in Ontario and Quebec sheep flocks during 2012 and 2013 (M.A. Drebot, unpub. data). Furthermore, we demonstrated that *An. quadrimaculatus* mosquitoes are a competent vector for both CVV lineages and reassortants. The differential susceptibility between lineage 1 and lineage 2 suggest that *An. quadrimaculatus* mosquitoes might be actively involved in lineage 1 displacement in the northeast United States and can potentially increase the risk for spillover to humans in the region because lineage 2 is more infectious and more readily transmitted.

We isolated 4 reassortant strains that contained lineage 1 L segments and lineage 2 S and M RNA segments, and all came from counties in western New York. Reassortment is an evolutionary mechanism of segmented RNA viruses to exchange genetic information during co-infection of cells, which generates new genotypes and phenotypes (*33**,**34*). During reassortment, entire genes are exchanged among different viral strains or species by the swapping of segments, which confer major fitness advantages or disadvantages to the progeny virus (*34*). In the family *Peribunyaviridae*, reassortment events have occurred between virus lineages. Intraspecies, interlineage reassortment events were reported for Rift Valley fever virus, a phlebovirus and a mosquitoborne zoonotic virus that affects domestic animals and humans (*35*), and also for Crimean-Congo hemorrhagic fever virus (*33**,**36**,**37*), a highly infectious orthonairovirus transmitted by *Hyalomma* spp. ticks. Furthermore, interspecies reassortment also occurs. For example, reassortment among Bunymawera serogroup viruses has been documented with Ngari virus and Potosi viru*s* (*38**–**41*), among others. In addition, although segment reassortment among California serogroup viruses is infrequent (*42*), evidence of reassortment has been documented (*43**,**44*).

Earlier studies had demonstrated that genetic reassortment between members of the family *Peribunyaviridae* can occur in vitro in mosquito and mammal cells and in vivo in mosquitoes during a mixed infection and can produce viable new strains with major phenotypic changes in terms of infectivity and pathogenicity (*38**–**40*,*42*,*45**–**47*). Furthermore, the phenomenon of superinfection resistance might promote opportunities for segment reassortment between more distantly related viruses. However, co-infection by closely related viruses can occur only in cases in which the second virus infects rapidly after the first virus and before superinfection resistance becomes effective (*38*). In our study, 3 CVV reassortants were isolated from mosquitoes and 1 was isolated from a human, and all contained the CVV lineage 1 L segment and CVV lineage 2 S and M segments. In addition, reassortant mosquito isolates that contained the L RNA segment with CVV lineage 1 were more infectious for *An. quadrimaculatus* mosquitoes than the lineage 1 strains, suggesting a probable role of the S or M RNA segments of lineage 2 strain in mosquito infectivity.

The vector competence of *Ae. albopictus* mosquitoes for Potosi virus and the susceptibility of *An. gambiae* Giles mosquitoes for Ngari virus has been demonstrated (*48*,*49*). Among the reassortant strains tested in our study, only the human reassortant strain was not transmitted by *An. quadrimaculatus* mosquitoes despite persistent infection. This difference in phenotype was probably not caused by the viral titer in the infectious blood meal because the titer was only ≈0.5 log_10_ lower for the human reassortant strain. We suspect that difference might be caused by other factors involving the virus strain and mosquito species used in our study. Addressing the potential mechanisms involved in differential vector competence phenotypes observed in *An. quadrimaculatus* mosquitoes and evaluating the role of strain variation in host competence and pathogenicity will help to clarify the consequences of genetic variation and displacement of CVV.
